# Vigilância da morbidade materna no Brasil: contribuições para o debate 

**DOI:** 10.1590/0102-311XPT151123

**Published:** 2023-11-13

**Authors:** Rosa Maria Soares Madeira Domingues, Marcos Augusto Bastos Dias, Valéria Saraceni, Rejane Sobrino Pinheiro, Natália Santana Paiva, Cláudia Medina Coeli

**Affiliations:** 1 Instituto Nacional de Infectologia Evandro Chagas, Fundação Oswaldo Cruz, Rio de Janeiro, Brasil.; 2 Instituto Nacional de Saúde da Mulher, da Criança e do Adolescente Fernandes Figueira, Fundação Oswaldo Cruz, Rio de Janeiro, Brasil.; 3 Secretaria Municipal de Saúde do Rio de Janeiro, Rio de Janeiro, Brasil.; 4 Instituto de Estudos em Saúde Coletiva, Universidade Federal do Rio de Janeiro, Rio de Janeiro, Brasil.

O artigo *Morbimortalidade Materna no Brasil e a Urgência de um Sistema Nacional de Vigilância do* Near Miss *Materno*, de autoria de Ferreira et al. ^1^, aborda o importante tema da morbidade materna e propõe um sistema nacional de vigilância do *near miss* materno.

Desde 2011, a Organização Mundial da Saúde (OMS) recomenda o estudo da morbidade materna grave de forma complementar ao estudo do óbito materno, por apresentar os mesmos determinantes e frequência mais elevada, permitindo análises mais robustas ^2^. Embora a razão de mortalidade materna global seja elevada, óbitos maternos apresentam frequência baixa, principalmente no âmbito dos serviços de saúde e em localidades com baixo número de nascimentos.

No Brasil, 2.389 municípios brasileiros não registraram sequer um óbito materno no período de 2012 a 2020 ^3^, demonstrando que estratégias baseadas apenas na investigação dos óbitos maternos são insuficientes para a formulação de estratégias de promoção da saúde materna e de melhoria do cuidado obstétrico.

Entretanto, a melhor forma de fazer a vigilância da morbidade materna grave e do *near miss* materno é incerta. Os autores do artigo publicado em CSP ^1^ propõem duas possíveis abordagens: o uso do Sistema de Informações Hospitalares do Sistema Único de Saúde (SIH/SUS) e a implantação da notificação de casos no Sistema de Informação de Agravos de Notificação (SINAN).

O SIH/SUS é o único sistema de informações brasileiro que contém dados de morbidade obstétrica durante internações hospitalares. Embora seja um sistema formulado para o pagamento de internações, tem sido utilizado para a vigilância do *near miss* materno; porém, com limitações.

Nakamura-Pereira et al. ^4^, em estudo realizado no Rio de Janeiro, mostraram que a sensibilidade e o valor preditivo positivo do SIH/SUS foram baixos para a detecção de casos de *near miss* materno. Esse resultado é, de alguma forma, esperado, já que, dos 25 critérios de disfunção orgânica recomendados pela OMS para classificação de casos de *near miss* materno, apenas um (ressuscitação cardiopulmonar) apresenta correspondência direta com os diagnósticos e procedimentos disponíveis no SIH/SUS. Muitos critérios recomendados pela OMS necessitam de informações detalhadas, como resultados de exames laboratoriais, parâmetros clínicos e duração de sintomas e/ou intervenções, que não estão disponíveis no SIH/SUS. Para contornar essas limitações, os autores utilizam critérios aproximados, que muitas vezes não refletem uma disfunção orgânica, como pretendido pela definição da OMS. Como resultado, os valores encontrados nesses estudos de incidência de *near miss* materno utilizando o SIH/SUS ^4^^,^^5^ são três a quatro vezes superiores aos relatados em estudos que realizam coleta de dados em prontuários ^6^^,^^7^.

Como alternativa para as dificuldades de operacionalização dos critérios da OMS, alguns autores adotam outras definições ^8^^,^^9^, como os critérios de Mantel e Waterstone, contrariando o propósito original da OMS ao recomendar uma definição padronizada de casos de *near miss* materno, que é permitir a comparação dos resultados entre diversos serviços e países.

Já a segunda proposta, de implantação da notificação de casos de *near miss* materno no SINAN, não parece ser de fácil operacionalização. Além da inexistência de código na Classificação Internacional de Doenças (CID) para casos de *near miss* materno, já mencionada pelos autores ^1^, estima-se que 1% das gestantes seja caso de *near miss* materno, o que, no Brasil, representaria cerca de 20 mil casos por ano. Entretanto, para a identificação de casos de *near miss* materno, é preciso que sejam triados os casos de morbidade materna grave, para que entre eles sejam identificados os que atendem aos critérios de disfunção orgânica. Casos de morbidade materna grave são mais frequentes, estimados em 10% do total de gestações. Ou seja, seria necessário criar uma estrutura de vigilância dos cerca de 2 milhões de nascimentos para identificação de 200 mil casos de morbidade materna grave e posterior notificação dos casos de *near miss* materno. Portanto, embora possível, a notificação do caso de *near miss* materno exigiria uma estrutura complexa de vigilância das internações obstétricas.

Sem desmerecer as propostas apresentadas pelos autores, e reforçando a necessidade de implantação de um sistema de vigilância da morbidade materna grave, e não apenas do *near miss* materno, apresentamos duas alternativas. A primeira é a utilização do SIH/SUS para a vigilância da morbidade materna grave, utilizando a definição da OMS para as “*condições potencialmente ameaçadoras à vida (CPAV)*” ^2^. Para a classificação desses casos, a OMS propõe 26 critérios baseados em diagnósticos e procedimentos, a maioria deles com operacionalização direta no SIH/SUS. Apenas quatro critérios não podem ser utilizados, seja por falta de código CID e procedimentos correspondentes no SIH/SUS (placenta acreta, increta ou percreta; retorno à sala cirúrgica; intubação não anestésica) ou ausência de informações específicas (trombocitopenia < 100.000).

Em recente edital de ciência de dados em saúde da mulher, com financiamento do Conselho Nacional de Desenvolvimento Científico e Tecnológico (CNPq) e da Fundação Bill & Melinda Gates (Estados Unidos), foi efetuado o cálculo do indicador de morbidade materna grave para todo o Brasil, por município, para o período de 2012 a 2020, utilizando os critérios da OMS para as CPAV. Além da frequência desses casos, são apresentadas as principais causas de morbidade (hipertensão, hemorragia e infecção) e quatro indicadores de manejo (transfusão de hemoderivados, realização de procedimentos cirúrgicos, internação em unidade de terapia intensiva (UTI) e tempo de permanência maior do que sete dias). Os indicadores calculados são de acesso gratuito e estão disponíveis na página de Internet do Observatório Obstétrico Brasileiro (https://observatorioobstetrico.shinyapps.io/painel-vigilancia-saude-materna/). Esses dados já permitem a análise e monitoramento de morbidade materna grave em todo o país, sendo de grande relevância, principalmente para os cerca de 4 mil municípios brasileiros que não apresentam óbitos maternos regularmente.

A segunda alternativa seria a implantação de um Sistema de Informação Perinatal (SIP), a exemplo do sistema adotado pela Organização Pan-Americana da Saúde (OPAS) e já implementado em diversos países da América Latina. O SIP permitiria o monitoramento dos casos de *near miss* materno e morbidade materna grave ^10^, bem como de outros indicadores relacionados à assistência perinatal. Uma outra possibilidade seria a implantação de uma Autorização de Internação Hospitalar específica para as internações obstétricas (AIH obstétrica), integrada ao SIH/SUS já existente, o que permitiria a identificação correta de todas as internações obstétricas, assim como o monitoramento e a avaliação das práticas assistenciais e dos desfechos maternos e perinatais, incluindo casos de morbidade materna grave e *near miss* materno. A implantação de fichas específicas nos prontuários hospitalares, visando à identificação de casos de morbidade materna grave e *near miss* materno, facilitaria o registro de casos, já utilizada com sucesso em outros contextos ^11^.

A ampliação da vigilância da saúde materna, visando à redução da mortalidade materna, é uma necessidade no país. Esperamos que o debate iniciado por Ferreira et al. ^1^ contribua para a definição da melhor estratégia a ser adotada e sua efetiva implementação.
